# A Retrospective Study Evaluating Guideline Adherence of Neonatal Parenteral Nutrition in a Belgian Neonatal Intensive Care Unit

**DOI:** 10.7759/cureus.56654

**Published:** 2024-03-21

**Authors:** Truc-Doan Nguyen, Garmt Meers, Pieter-Jan Cortoos, Stephane Steurbaut, Filip Cools

**Affiliations:** 1 Department of Neonatology, University Hospital Brussels, Brussels, BEL; 2 Department of Hospital Pharmacy, University Hospital Brussels, Brussels, BEL; 3 Faculty of Medicine and Pharmacy, Vrije Universiteit Brussel, Brussels, BEL

**Keywords:** practice patterns, guideline adherence, newborn, infant, premature birth, parenteral nutrition

## Abstract

Introduction

Clinical nutrition for preterm and critically ill neonates remains a challenge. Preterms are often hemodynamically and metabolically compromised, which limits infusion volumes of nutrients and hinders achieving recommended nutrient intakes. While guidelines provide recommended ranges for parenteral nutrition (PN) intakes, they generally recommend enteral nutrition as soon as possible. Thus, in clinical practice, gradually increasing EN intakes complicates assessments of PN guideline adherence. Via a pragmatic approach, we assessed adherence to PN recommendations for macronutrients and energy as stated in the 2018 guidelines of the European Society for Paediatric Gastroenterology, Hepatology and Nutrition (ESPGHAN).

Methods

In this retrospective study, we assessed the nutrition of preterm and critically ill term neonates from the neonatal intensive care unit of the University Hospital Brussels. We analyzed intakes for the first week of life, in which critically ill neonates at our center usually receive the majority of nutrients via PN. The PN-based provision of macronutrients and energy was analyzed descriptively in relation to the ESPGHAN 2018 recommendations.

Results

Macronutrients and energy provision gradually increased until they reached recommended or targeted values. Compared to term neonates, energy and lipid provision for preterms increased faster, while amino acid provision exceeded the ESPGHAN 2018 recommendations.

Conclusions

This study adds clinical practice data to the severely understudied field of the ESPGHAN 2018 PN guideline compliance. Using a pragmatic assessment of our nutrition protocols, we found the need to reduce the amount of amino acids per kg body weight per day to meet guideline recommendations.

## Introduction

Clinical nutrition in preterm and critically ill neonates remains a challenge in daily practice, with substantial evidence gaps, e.g., the optimal glucose and lipid intakes for maximizing protein accretion [1]. This might explain why observational studies showed that nutrition in critically ill preterm and term neonates fails to reach recommended intake levels for parenteral nutrition (PN) intakes [2].

Neonates require energy above the resting energy expenditure for growth. Resting energy expenditure in healthy preterm infants has been described in the range of 35-55 kcal/kg body weight (BW)/d during the first two weeks after birth, increasing to about 70 kcal/kg BW/d one month after birth [2]. Very premature neonates require an additional 50-70 kcal/kg BW/d to reach intrauterine growth rates [2]. Providing adequate nutrition to preterm and critically ill neonates enables them to reach their respective growth potentials [2]. As preterm and critically ill neonates are often hemodynamically and metabolically compromised, these requirements have to be met with small infusion volumes [3]. Due to these small infusion volumes, nutritional supply often fails to reach the required levels, resulting in cumulative energy and amino acid deficits [3,4]. Studies have reported the associations between insufficient nutrition of neonates after birth and disadvantageous outcomes for weight gain [5], neuronal development [6], and retinopathy of prematurity [7].

Studies have demonstrated that overcoming these obstacles to providing adequate nutrition for preterm and critically ill neonates can result in substantial gains. A Cochrane meta-analysis of higher vs. lower amino acid provision by PN for predominantly very low birth weight (VLBW, <1,500 g) or very preterm neonates (<28 weeks gestational age (GA)) found that higher amino acid provision was associated with positive effects on growth and prevention of postnatal growth failure [8]. More recent studies showed a linear association between increased energy and macronutrient provision during the first weeks and increased weight and length gain in VLBW infants [9,10]. In the long term, the supply of calories and protein during the first week after birth has a direct impact on the Mental Development Index during the subsequent 18 months in extremely low birth weight (<1,000 g) neonates [11]. Increased energy intake in the first week after birth was associated with increased fat-free mass throughout early infancy, independent of comorbidities in VLBW preterms [12].

The European Society for Paediatric Gastroenterology, Hepatology and Nutrition (ESPGHAN) updated its guidelines for neonatal PN in 2018. These guidelines provide guidance on daily energy and macronutrient requirements based on the GA and day of life [13-16]. In clinical practice, the ESPGHAN PN recommendations have to be aligned with both the overall energy provision and the clinical situation, e.g., acute illness or fluid restrictions [16].

While guideline recommendations apply solely to PN, clinicians gradually introduce enteral nutrition (EN) as soon as possible. This complicates observational analyses of PN guideline adherence. While the ESPGHAN also released EN guidelines for preterm infants in 2022 [17], the society currently provides no recommendations for concomitant PN and EN macronutrient intakes. Thus, it is methodologically difficult to assess the adherence to PN or EN guidelines outside of total PN or total EN situations, unless compromises are made. For instance, Boscarino et al. compared the total energy intake to the ESPGHAN 2018 PN recommendations as long as PN provided ≥70% of the total energy [18]. Brinkis et al. decided to evaluate the combined PN and EN intakes against the ESPGHAN 2022 EN guidelines, which seems appropriate for their four-week assessment period, given that EN intakes were the main source of nutrition starting from the second week [19]. Nonetheless, as long as critically ill infants receive nutrients mainly via PN, it is important to analyze the feasibility of and adherence to the ESPGHAN PN guideline recommendations in a real-world situation in order to adapt nutrition protocols.

Surveys found adherence to guidelines for PN in neonates to vary widely between European centers, and provision was often inadequate [20,21]. However, very few studies have compared the 2018 ESPGHAN guideline recommendations with current routine practice in the parenteral feeding of neonates [22,23]. Here, we report a pragmatic approach to evaluate the ESPGHAN 2018 guideline adequacy of PN with individual macronutrient solutions, considering gradually increasing EN after birth. This approach was evaluated in a large, consecutively documented cohort of neonates admitted to the neonatal intensive care unit (NICU) of the University Hospital Brussels (UZ Brussel). Additionally, we assessed the occurrence of PN-associated complications. These analyses were used to determine if and how the UZ Brussel would need to adapt their practices to meet the ESPGHAN 2018 recommendations.

## Materials and methods

Study design and patient population

This retrospective cohort study was conducted at UZ Brussel, Brussels, Belgium and involved infants hospitalized in its NICU between January 1, 2017 and August 31, 2019. PN was started upon admission to the NICU for all neonates for whom the treating physicians considered enteral feeding to be insufficient to meet nutritional goals.

Inclusion criteria were, regardless of GA, (1) newborn infants admitted to the NICU at the UZ Brussel from January 1, 2017 until August 31, 2019; (2) BW >500 g; (3) receiving PN for a minimum of 72 hours after admission to the NICU; and (4) at least one-third of the patient’s total fluid intake was delivered via PN. Exclusion criteria were (1) clinical conditions for which PN was either contraindicated or restricted: inborn errors of metabolism and renal or hepatic compromise; (2) decision for withdrawal of intensive care during the phase of PN; (3) transfer to another hospital during the phase of PN; and (4) early postnatal death (within the first 48 hours of life).

This study was approved by the Medical Ethics Committee at UZ Brussel with approval number B.U.N. 143201941266 (date of approval: September 25, 2019).

Nutrition, variables, and data sources

The primary outcome was the total nutrition provision for energy (kcal/kg/d), amino acids (g/kg/d), lipids (g/kg/d), and glucose (mg/kg/min). Enteral feeding was introduced as soon as possible after birth, at the discretion of the treating physician. In practice, all patients received some enteral feeding a few days after PN started. PN was gradually reduced with increasing volumes of enteral feeding and stopped if the enteral intake reached 120-130 ml/kg. Enteral feed was preferentially own mother’s milk. If the EN volume exceeded 100 ml/kg/d, the mother’s milk was fortified with Human Milk Fortifier (Nestlé Belgilux SA, Brussels, Belgium) at a concentration of 3-4%, depending on the patient’s weight. If the mother’s milk was unavailable, formula milk was used according to the BW of the patient (<1,700 g preterm formula, 1,700-2,500 g ex-preterm formula, and >2,500 g term formula).

Similar to a study by Brinkis et al., we combined intakes from both PN and EN to assess the adherence of the nutrient provision to guideline recommendations and targeted intakes [19]. While PN and EN may differ in the nutrient and energy amounts provided per volume, we pragmatically considered both PN and EN volume-equivalent in their nutrient and energy provision for two reasons. First, in clinical practice at the NICU at the UZ Brussel, PN provides most of the macronutrients in the first week, while EN intakes only gradually move from trophic feeding (<20 ml/kg/day) to substantial provision of nutrients. Second, while the ESPGHAN 2018 PN guidelines outline the differences between PN and EN energy delivery, they acknowledge that the inclusion of these differences in clinical practice is difficult. This is reflected in the use of Atwater factors to calculate energy intakes from both PN and EN, although they do not factor in the route of delivery [1]. Trophic feeding was not taken into account for this approach.

The PN solutions were compounded individually for each patient at the hospital pharmacy. Our established nutrition protocol consisted of a combination of mainly glucose and amino acid solutions during the first days after birth that was complemented with a lipid emulsion from the second or third day on. The used commercial products were the amino acid solution Vaminolact (Fresenius Kabi NV/SA, Schelle, Belgium) or alternatively Primene (Baxter Belgium BVBA, Braine-l’Alleud, Belgium), glucose 10% or 50% (Baxter Belgium BVBA), and the lipid emulsion SMOFlipid (Fresenius Kabi NV/SA). Intake data were recorded daily for the first week after birth. The intake data were described in relation to the respective the ESPGHAN 2018 recommendations [13-16]. In the case of lipids, our center aimed for intakes of 3 g/kg/d to avoid exceeding the recommended maximum of 4 g/kg/d [15].

The secondary outcome was the incidence rate of PN-related complications, namely hyperglycemia (serum glucose levels of 150-200 mg/dL), diagnosis of PN-associated liver disease, central line-associated bloodstream infection (CLABSI), thrombotic complications, metabolic bone disease, and the proportion of patients with more than one complication. Outcomes were analyzed for all GA subgroups.

Macronutrients supplied via PN were calculated using the Nutriscript software (Fresenius Kabi NV/SA). Thus, data on daily PN intakes for energy and macronutrients were derived from the prescriptions (fluid volumes as previously described in Batteux et al. [23]). All other parameters were extracted from electronic medical files, including the start of PN, stop of PN, and GA, as well as birth data for weight and length.

Statistical methods

For descriptive statistics, baseline data were summarized via medians and interquartile ranges. Adherence to the ESPGHAN 2018 guideline recommendations was assessed graphically for all three GA groups by plotting the mean ± SD of the respective nutrient or energy provision against the respective guideline recommendations. PN-related complications were summarized as counts and percentages. Missing data were not imputed.

## Results

Patient characteristics

In total, 201 neonates were eligible for the study period (Table 1). Of the 201 neonates enrolled in this study, 110 (54.7%) were very preterm (<32 weeks GA), and 65 (32.3%) were preterm (32-36 weeks GA). An appropriate length for the GA was present in the majority of neonates (191 patients, 95.0%), and delivery by cesarean section took place in nearly three-quarters of cases (145 patients, 72.1%). Most of the neonates were born in the NICU of the UZ Brussel (163 patients, 81.8%). For the whole cohort, the median Apgar score after five minutes was 9 (IQR: 7-9; range: 1-10).

**Table 1:** Summary of patient characteristics Length appropriateness was derived from the Z-score at birth, with ≤-2 as small, between -2 and 2 as appropriate, and ≥2 as large. GA, gestational age

Parameter	N (%)	Median	IQR
Sex			
Male	112 (55.7)	-	-
Female	89 (44.3)	-	-
Birth weight (g)		1,585	1,150–2,100
<1,000 g	40 (19.9)	-	-
1,000–1,499 g	52 (25.9)	-	-
1,500–2,499 g	67 (33.3)	-	-
≥2,500 g	42 (20.9)	-	-
GA (weeks)		31.7	29.1–33-6
<28 weeks	33 (16.4)	-	-
28–31 weeks	77 (38.3)	-	-
32–36 weeks	65 (32.3)	-	-
≥37 weeks	26 (12.9)	-	-
Length appropriateness			
Small for GA	8 (4.0)	-	-
Appropriate length for GA	191 (95.0)	-	-
Large for GA	2 (1.0)	-	-
Other characteristics			
Apgar score (after five minutes)	201 (100)	9	7.25–9.00
Cesarean section	145 (72.1)	-	-
Multiple births	55 (27.4)	-	-
Inborn at the UZ Brussel	163 (81.8)	-	-

As Table 2 shows, prematurity was the most common indication for NICU admission by a huge margin (77.5%), followed by respiratory distress syndrome (8.5%) and perinatal asphyxia (6.0%).

**Table 2:** Indications for admission to the NICU of the UZ Brussel Percentages may not add up to 100 due to rounding. NICU, neonatal intensive care unit

Indication	N (%)
Prematurity	155 (77.1)
Respiratory distress syndrome	17 (8.5)
Perinatal asphyxia	12 (6.0)
Congenital abnormality (gastrointestinal)	6 (3.0)
Congenital abnormality (non-gastrointestinal)	3 (1.5)
Other	7 (3.5)
Missing information	1 (0.5)

Intake of energy and macronutrients

**Figure 1:**
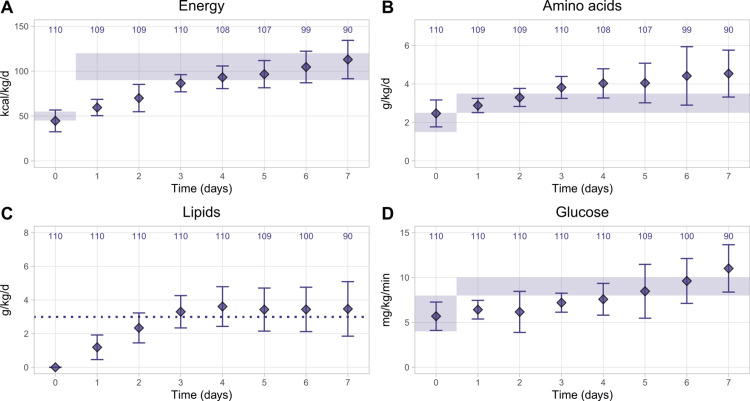
Calculated mean nutrient provision in very preterm infants (GA <32 weeks) over time for (A) energy, (B) amino acids, (C) lipids, and (D) glucose Data points show the mean ± SD. The numbers on top show patient numbers. Shaded areas show the minimum and maximum intakes for energy, amino acids, and carbohydrates recommended by the ESPGHAN 2018 guidelines, and the dotted line shows the lipid intake target used in the UZ Brussel. GA, gestational age; ESPGHAN, European Society for Paediatric Gastroenterology, Hepatology and Nutrition [13,14,16]

The means of providing energy and macronutrients met the ESPGHAN recommendations or target values at different times, depending on the GA group. For very preterm infants, it took about four days until the average energy intake reached the ESPGHAN recommendations of 90-120 kcal/kg/d (Figure 1A) [16]. In contrast, the amino acid provision was close to recommendations for the first two days but then exceeded them for the rest of the follow-ups, peaking at 4.54 ± 1.22 g/kg/d on the seventh day (Figure 1B). Lipid provision gradually increased from no intakes until they approximated the targeted dose of 3 g/kg/d starting after D3 until the end of the follow-up (Figure 1C). Glucose provision gradually increased after D2 (Figure 1D). It approximated guideline recommendations of 8-10 mg/kg/min [14] after D5 (8.53 ± 1.26 mg/kg/min).

**Figure 2:**
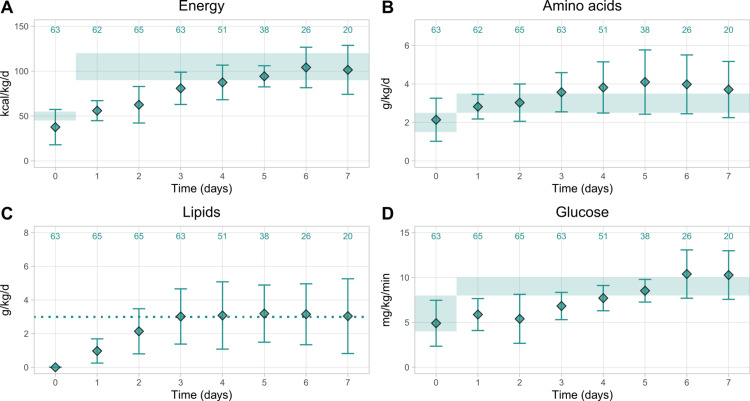
Calculated mean nutrient provision in preterm infants (GA 32–36 weeks) over time for (A) energy, (B) amino acids, (C) lipids, and (D) glucose Data points show the mean ± SD. The numbers on top show patient numbers. Shaded areas show the minimum and maximum intakes for energy, amino acids, and carbohydrates recommended by the ESPGHAN 2018 guidelines, and the dotted line shows the targeted lipid intake in the UZ Brussel. GA, gestational age; ESPGHAN, European Society for Paediatric Gastroenterology, Hepatology and Nutrition [13,14,16]

Figure 2 shows the mean nutrient provision for preterm infants. Energy provision increased slowly until it reached recommended levels on D5 (Figure 2A), while amino acid provision started in the recommended range and exceeded it slightly after D4, peaking on the fifth day (4.10 ± 1.67 g/kg/d; Figure 2B). Lipid provision increased for the first three days and then remained close to the targeted intake of 3 g/kg/d for the first week (Figure 2C). For glucose, provision increased gradually until it reached the recommended levels after D5 (Figure 2D).

**Figure 3:**
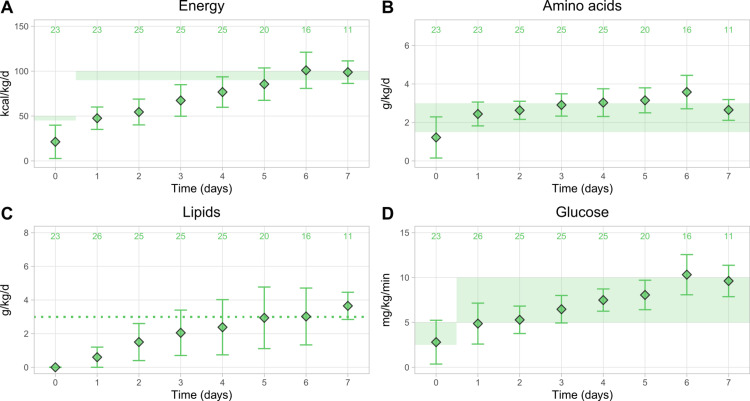
Calculated mean nutrient provision in term infants (GA >36 weeks) over time for (A) energy, (B) amino acids, (C) lipids, and (D) glucose Data points show the mean ± SD. The numbers on top show patient numbers. Shaded areas show the minimum and maximum intakes for energy, amino acids, and carbohydrates recommended by the ESPGHAN 2018 guidelines, and the dotted line shows the targeted lipid intake in the UZ Brussel. GA, gestational age; ESPGHAN, European Society for Paediatric Gastroenterology, Hepatology and Nutrition [13,14,16]

Figure 3 shows the mean nutrient provision for term infants. Provision of energy (Figure 3A), lipids (Figure 3C), and glucose (Figure 3D) increased gradually and nearly linearly during the first week. Energy and lipid provision met the recommended or targeted levels after D6, while glucose provision was close to or in the recommended intake range throughout the first week. Amino acid provision was in the recommended range after D1 (Figure 3B). It increased slowly until it slightly exceeded recommendations, peaking at 3.58 ± 0.87 g/kg/d on the sixth day.

PN-related complications

Table 3 summarizes the number of PN-related complications. Among the 201 complications, the most prevalent ones were CLABSI (23, 11.4%) and metabolic bone disease (22, 10.9%). Most of the PN-related complications occurred in very preterm infants.

**Table 3:** Summary of PN-related complications CLABSI, central line-associated bloodstream infection; GA, gestational age; PN, parenteral nutrition; PNALD, parenteral nutrition-associated liver disease

PN-related complications	N (%)	N, <32 weeks GA	N, 32–36 weeks GA	N, >36 weeks GA
Hyperglycemia	13 (6.5)	12	0	1
PNALD	5 (2.5)	5	0	0
CLABSI	23 (11.4)	18	3	2
Thrombotic complication	3 (1.5)	2	0	1
Metabolic bone disease	22 (10.9)	18	3	1
More than one complication	20 (10.0)	19	0	1

## Discussion

Generally, in all GA groups, the provision of energy and macronutrients gradually increased during the first week until they reached recommended or targeted values. For all age groups, all provisions were at least close to recommendations after three to five days. Further, there are three major differences between term versus very preterm or preterm infants. First, for term infants, it took about one to two days longer until recommended levels for energy were reached, conceivably due to the relatively lower lipid provision. Second, after one day, term infants had amino acid intakes in line with or close to the ESPGHAN 2018 recommendations, while intakes for (very) preterms exceeded recommendations from the third or fourth day onward. Third, for term infants, recommendations for glucose were met from the first day on, while it took about five days for (very) preterms to reach them. Taken together, these differences imply that for very preterm and term infants, the provision of energy and lipids was increased at a faster rate and that amino acid provision exceeded the ESPGHAN 2018 recommendations. This finding demonstrates that practices at the UZ Brussel were still largely following the ESPGHAN 2005 guidelines at the time of the study, which recommended a higher maximum amino acid intake of 4 g/kg/d [24].

Based on our results, the PN regimen in the Nutriscript program was adapted to follow the ESPGHAN 2018 guidelines more closely, with amino acid provision set to 2.0 g/kg/d (previously: 2.5 g/kg/d) for both preterm and term neonates on day 1, and on subsequent days to an upper limit of 2.5 g/kg/d for term and 3.5 g/kg/d for preterm neonates (previously: upper limits of 4.0 and 3.5 g/kg/d, respectively). Due to their well-established use and safety, the composition of the nutrient provision was left unchanged.

Generally, the observed gradual increase of energy and macronutrient provision is well known from the literature [25-27] and recommended by the ESPGHAN 2018 guidelines for carbohydrates [14] and, for the first two days, for energy [16] and amino acids [13] as well. The phases of “underfeeding” in the days after birth reflect the need to balance the nutritional needs of the newborns with the risk of metabolic complications and to restrict fluid intake, especially in very premature newborns [2]. This is most obvious in the glucose provision for preterms, which reached guideline recommendations later than in term infants because clinicians had to balance the need for energy against the risk of hyperglycemia [14]. Patients at the NICU of the UZ Brussel receive EN as soon as possible after birth, and this transition phase is regarded as the most critical time for the achievement of nutritional goals [28]. This is another reason for the careful increase in energy and macronutrient provision.

The rationale behind the higher and faster-increasing nutrient provision for preterms is based on the well-documented benefits of this approach, as the energy and macronutrient intakes in the first week are crucial for the development of preterms [25,28]. For instance, Wang et al. observed for extremely low birth weight (<1,000 g) and VLBW infants that for each additional day it took to reach maximum energy and glucose intake recommendations, the probability of an appropriate weight gain outcome decreased by 5.6% and 6.1%, respectively [25]. Westin et al. found that an earlier and more aggressive PN improved energy, protein, and lipid provision during the first days of life for preterm infants of GA <27 weeks [29]: minimal energy requirements of 85-95 kcal/kg/d on days 4-6 were achieved for nearly 90% of the infants in their study. An intake of 3.5 g/kg/d protein was reached for >50% of the patients within six days after birth.

Combining both PN and EN poses significant problems in assessing whether nutritional recommendations are actually met. As EN and PN are inherently different and have different goals, our approach is an approximation and certainly not suited for individual patient assessments, which is the main limitation of this study. Amino acids, lipids (with medium chain triglycerides), and glucose may provide about 10% less kcal/g than proteins, lipids, and more complex carbohydrates from EN, respectively [16], so it is possible that actual energy intakes were slightly higher than calculated. Therefore, the approach for the detection of general trends in nutritional care practice suggests changes where needed. Other centers may benefit as well by looking back on their practice using this pragmatic approach. Besides the potential single-center bias, our analyses are limited by missing data, as for any retrospective study. Nonetheless, this study included over 200 neonates, which is relatively large compared to similar studies on current the ESPGHAN guideline adherence [19,30]. The large cohort together, with a study period of more than 2.5 years, indicates that our population is highly representative for our hospital in particular and for other centers in general.

## Conclusions

This study adds clinical practice data to the understudied field of the ESPGHAN 2018 PN guideline compliance. By using a pragmatic approach to determine if the energy and macronutrient provision in the NICU of the UZ Brussel met the ESPGHAN 2018 recommendations, we could determine that targets for total energy and lipids were met earlier in preterms (GA ≤36 weeks) than in term infants, while preterms tended to have higher amino acid provision above the ESPGHAN recommendations. Consequently, we substantially lowered the parameters for amino acid provision in our Nutriscript program to better reflect the ESPGHAN 2018 guideline recommendations. Due to its well-established use and safety, the nutrient provision continues to be composed of Vaminolact or Primene, SMOFlipid, and glucose. Prospective follow-up studies will assess how these protocol changes affect guideline adherence and growth outcomes.
